# Tumor cell-imposed iron restriction drives immunosuppressive polarization of tumor-associated macrophages

**DOI:** 10.1186/s12967-021-03034-7

**Published:** 2021-08-13

**Authors:** Jia-Lei Sun, Ning-Ping Zhang, Ru-Chen Xu, Guang-Cong Zhang, Zhi-Yong Liu, Weinire Abuduwaili, Fu Wang, Xiang-Nan Yu, Xuan Shi, Guang-Qi Song, Hao Wu, Tao-Tao Liu, Xi-Zhong Shen, Bin Deng, Shu-Qiang Weng, Ling Dong, Ji-Min Zhu

**Affiliations:** 1 Department of Gastroenterology and Hepatology, Zhongshan Hospital of Fudan University, 180 Fenglin Rd., Shanghai, 200032 China; 2 Shanghai Institute of Liver Disease, Shanghai, China; 3 Key Laboratory of Medical Molecular Virology, Shanghai Medical College of Fudan University, Shanghai, China; 4 Department of Gastroenterology, The Affiliated Hospital of Yangzhou University, Yangzhou, 225001 China

**Keywords:** Hepatocellular carcinoma, Iron metabolism, Tumor-associated macrophages, Transferrin receptor, Tumor immunology

## Abstract

**Background:**

Tumor-associated macrophages (TAM) are immunosuppressive cells that contribute to impaired anti-cancer immunity. Iron plays a critical role in regulating macrophage function. However, it is still elusive whether it can drive the functional polarization of macrophages in the context of cancer and how tumor cells affect the iron-handing properties of TAM. In this study, using hepatocellular carcinoma (HCC) as a study model, we aimed to explore the effect and mechanism of reduced ferrous iron in TAM.

**Methods:**

TAM from HCC patients and mouse HCC tissues were collected to analyze the level of ferrous iron. Quantitative real-time PCR was used to assess M1 or M2 signature genes of macrophages treated with iron chelators. A co-culture system was established to explore the iron competition between macrophages and HCC cells. Flow cytometry analysis was performed to determine the holo-transferrin uptake of macrophages. HCC samples from The Cancer Genome Atlas (TCGA) were enrolled to evaluate the prognostic value of transferrin receptor (TFRC) and its relevance to tumor-infiltrating M2 macrophages.

**Results:**

We revealed that ferrous iron in M2-like TAM is lower than that in M1-like TAM. In vitro analysis showed that loss of iron-induced immunosuppressive M2 polarization of mouse macrophages. Further experiments showed that TFRC, the primary receptor for transferrin-mediated iron uptake, was overexpressed on HCC cells but not TAM. Mechanistically, HCC cells competed with macrophages for iron to upregulate the expression of M2-related genes via induction of HIF-1α, thus contributing to M2-like TAM polarization. We further clarified the oncogenic role of TFRC in HCC patients by TCGA. TFRC is significantly increased in varieties of malignancies, including HCC, and HCC patients with high TFRC levels have considerably shortened overall survival. Also, TFRC is shown to be positively related to tumor-infiltrating M2 macrophages.

**Conclusions:**

Collectively, we identified iron starvation through TFRC-mediated iron competition drives functional immunosuppressive polarization of TAM, providing new insight into the interconnection between iron metabolism and tumor immunity.

**Supplementary Information:**

The online version contains supplementary material available at 10.1186/s12967-021-03034-7.

## Introduction

Macrophages, highly heterogeneous immunocytes, play crucial roles in maintaining homeostasis and physiological processes [1]. This cluster of cells exhibits a spectrum of functional, metabolic, and phenotypic characteristics inducing a shift from a pro-inflammatory to an anti-inflammatory pattern in response to environmental stimuli [2]. Microbe-associated molecular patterns (e.g., LPS) and Th1 cytokines (e.g., IFN-γ, IL-2, and TNF-α) activate macrophages into an M1 inflammatory state, resulting in upregulated expression of inflammation-related genes (e.g., *iNOS*, *CXCL9*, and *CXCL10*) [3]. In contrast, Th2 cytokines (e.g., IL-4, IL-10, and CSF1) polarize cells to an M2 phenotype with immunoregulatory and anti-inflammatory functions, resulting in elevated expression of genes such as *ARG1* (arginase-1), *PD-L1* (programmed cell death-ligand 1), and *VEGFA* (vascular endothelial growth factor type A) [3, 4]. Therefore, it can be understood that macrophages regulate immune homeostasis in a tissue-specific and context-dependent manner. However, whether tissue environment polarizes subsets of specific macrophages remains unknown.

Tumor-infiltrating macrophages, also known as tumor-associated macrophages (TAM), are a predominant cellular part of the tumor microenvironment (TME) in solid malignancies [5]. The proportion of TAM is positively correlated with poor diagnosis and prognosis of immunotherapy [6]. Accumulating evidence suggests that TAM acquires an inhibitory M2-like phenotype to negatively regulate cytotoxic function of effector immune cells within the TME. Therefore, it disrupts anti-tumor immunity and leads to tumor immune evasion [7–9]. Besides known cytokines that activate TAM to an M2-like phenotype, tumor-derived metabolites are also critical factors that mediate TAM's immunosuppressive polarization. Tumor-derived lactate, a byproduct of tumor glycolysis, leads to M2-like TAM polarization by activating HIF-1α and subsequent inducing ARG1 and VEGFA [10]. Lactate can also inhibit ATP6V0d2, a macrophage-specific isoform of the vacuolar ATPase subunit, to promote HIF-2α-mediated M2-like TAM polarization [11]. In another study, tumor-derived succinate generated from succinyl-CoA by TCA-cycle enzyme succinyl-CoA synthetase drives M2-like TAM polarization to promote cancer metastasis via the PI3K–HIF-1α axis [12]. These findings suggest that tumor metabolism is closely associated with M2-like TAM polarization. Thus, a better understanding of how tumor-derived metabolites regulate TAM may provide novel anti-tumor strategies.

Iron metabolism is essential for physiological cellular processes, including mitochondrial respiratory, cell cycle, and detoxification [13]. Being absorbed in the duodenum, ferric iron (Fe^3+^) from the diet is loaded onto transferrin (TF) and then taken up by cells through transferrin receptor (TFRC)-mediated endocytosis [14, 15]. Intracellular iron is then reduced into ferrous iron (Fe^2+^) and transferred by metal-ion transporter 1 (DMT1) to the utilizable labile iron pool (LIP) [16]. The ferrous iron in LIP is highly active and acts as a primary functional iron [17]. Excess iron can either be stored in ferritin or be exported from cells by solute carrier family 40 member 1 (SLC40A1), an iron exporter [18, 19]. Being an essential component of cellular homeostasis, iron metabolism is also critical for inflammation processes [20]. For example, iron can drive helper T cell pathogenicity by promoting pro-inflammatory cytokine production [21]. Meanwhile, emerging evidence suggests reciprocal interconnections between iron homeostasis and macrophage-mediated inflammatory processes [20]. It has been reported that macrophage-derived lipocalin-2 (LCN2), a high-affinity iron carrier protein, links to renal recovery during sepsis-induced kidney damage [22]. Another study indicated that Iron-laden macrophages show an enhanced M1 phenotype with elevated TNF production [23]. In contrast, iron loading of macrophages leads to reduced M2 markers in the presence of IL-4 [24]. These observations suggest the essential role of iron in macrophage polarization. Considering the aberrant iron metabolism in cancer cells, it remains elusive whether cancer cells could influence the TAM polarization.

In this study, using hepatocellular carcinoma (HCC) as a study model, we examined the expression level of ferrous iron in TAM from HCC patient samples and murine model, and explored the effect and mechanism of reduced ferrous iron in TAM. Together, these findings identified that the TF/TFRC axis-mediated iron uptake of HCC cells directly competes for iron with macrophages to reduce iron in TAM and their M2 polarization.

## Materials and methods

### Cell culture

Mouse HCC cell line Hepa1-6 and mouse macrophage RAW264.7 were obtained from the cell bank of the Chinese Academy of Science (Shanghai, China). Hepa1-6 cells and RAW264.7 cells were cultured in Dulbecco’s modified Eagle’s medium (DMEM) (Hyclone, USA) supplemented with 10% fetal bovine serum (Sigma, USA). All cells grew at 37 °C and 5% CO_2_ in a humidified incubator (Thermo Fisher Scientific, USA).

### Reagents

Deferasirox (S1712) and ciclopirox (S2528) were purchased from Selleck, USA. FITC-labeled holo-transferrin was from Jackson ImmunoResearch, USA. Ferric citrate and lactate were from Sangon Biotech, China.

### Preparation of mouse peritoneal macrophages

To obtain bone marrow-derived macrophages, bone marrow cells were isolated from mouse tibia and femur, and then cultured in 10 cm dish with DMEM containing 50 ng/mL recombinant mouse macrophage colony-stimulating factor (M-CSF; Novoprotein, China). After 2 days of culture, the supernatant was replaced with DMEM containing 50 ng/mL M-CSF. The cells were allowed to grow for additional 4–5 days. The adherent macrophages were collected for further experiments.

For isolation of mouse peritoneal macrophages, 4% Thioglycolate (BD bioscience, USA) was intraperitoneally injected into 6-week-old C57BL/6 mice. 4 days later, mice were sacrificed, peritoneal macrophages were collected by peritoneal cavity lavage with 8–10 mL DMEM. After centrifugation at 5000 rpm for 5 min, macrophages were resuspended for further experiments.

### Bioinformatics analysis

TFRC expression between tumor and non-tumor tissues from various tumors obtained from The Cancer Genome Atlas (TCGA) was assessed using the ‘Gene_DE’ module in Tumor Immune Estimation Resource (TIMER; http://timer.cistrome.org/).

The Gene Expression Profiling Interactive Analysis (GEPIA) database was used to evaluate the prognostic value of TFRC in TCGA-LIHC (Liver Hepatocellular Carcinoma) cohorts. Kaplan–Meier analysis for overall survival (OS) was performed in a total of 364 HHC patients using median TFRC expression as the cut-off. The correlation of TFRC and M1 or M2 signature genes was determined using the ‘Correlation’ module.

The assessment of tumor-infiltrating immune cells was performed using the CIBERSORT database (https://cibersort.stanford.edu/). Briefly, a leukocyte gene signature matrix containing 547 genes was used to characterize immune cell subtypes from 22 human species. RNA-seq V2 data of LIHC patients were obtained from the Cbioprotal (http://www.cbioportal.org/). The RNA-seq data were applied to the CIBERSORT algorithm, and the proportions of different immune cell populations were acquired using the ‘Cell Fraction Analysis’ module.

### Flow cytometry (FACS)

Cells with indicated treatment were harvested by trypsinization, resuspended in cold PBS, and stained with antibodies against CD206-FITC (Thermo, USA) PD-L1-PE (Thermo, USA) for 30 min on ice. After washes with PBS two times, cells were resuspended in PBS and analyzed using a Beckman Gallios flow cytometer. The data were analyzed by FlowJo V10 software (FlowJo LLC, USA).

For human macrophages, fresh tumor samples from 6 HCC patients, who underwent curative resection at Zhongshan Hospital, Fudan University, were collected for analysis in our study. Tumor tissues were minced and excised into small pieces followed by incubation in DMEM containing 1 mg/mL collagenase IV (Sigma, USA) and 10^–3^ U/L DNase I (Invitrogen, USA) for 1 h. After lysed, single-cell suspensions were stained with the following reagents and antibodies: Live&Dead APC-cy7 (Invitrogen, USA), CD45-BV510 (BD bioscience, USA), CD11b-APC (Invitrogen, USA), CD11c-BV421 (BD bioscience, USA), MHC-II-FITC (Invitrogen, USA), and FerroOrange (Dojindo, Japan) for 30 min. Cells were washed 2 times with PBS and analyzed by FACS. M1-like human TAM including Live&Dead^−^, CD45^+^, CD11b^+^, CD11c^−^, and MHC-II^+^. M2-like human TAM including Live&Dead^−^, CD45^+^, CD11b^+^, CD11c^−^, and MHC-II^−^. All clinical specimens were collected from patients enrolled after obtaining informed consent following a protocol approved by the Ethics Committee of Zhongshan Hospital, Fudan University.

For mouse macrophages, Hepa1-6 cells (5 × 10^6^) were first subcutaneously implanted into right flanks of C57BL/6 mice (Charles River, China). After 2 weeks, mice were sacrificed, and the tumors were dissected. Single-cell suspensions were prepared as described above and stained with the following reagent and antibodies: Live&Dead Fluor 506 (Invitrogen, USA), CD45-BV395 (BD bioscience, USA), CD11b-PerCP-cy5.5 (Invitrogen, USA), Gr-1-APC (Invitrogen, USA), F4/80-PE (Invitrogen, USA), MHC-II-FITC (Invitrogen, USA), and FerroOrange (Dojindo, Japan) for 30 min. Cells were washed 2 times with PBS and analyzed by FACS. M1-like mouse TAM including Live&Dead^−^, CD45^+^, CD11b^+^, Gr-1^−^, F4/80^+^, and MHC-II^+^. M2-like mouse TAM including Live&Dead^−^, CD45^+^, CD11b^+^, Gr-1^−^, F4/80^+^, and MHC-II^−^.

Mean fluorescence intensity (MFI) was used to determine the expression level of indicated proteins. The MFI of control was normalized to 1, and the fold changes in each group were calculated relative to the control.

### Plasmids, small interfering RNA (siRNA), and transfection

For *Tfrc* knockdown in Hepa1-6 cells, lentiviruses were generated by transfecting 10 μg plasmids PGMLV-shNT, PGMLV-shTfrc together with 5 μg psPAX2 and 7.5 μg pMD2.G into 293 T cells reaching 50% confluency in a 6-well plate. 72 h after transfection, the supernatants were collected, centrifuged and the viral-containing supernatants were used to infect Hepa1-6 cells for 8 h. After that, the medium was removed, and a new medium with 5 μg/mL puromycin was replaced to select for stably *Tfrc* knockdown in Hepa1-6 cells.

For *Hif-1α* and *Hif-2α* knockdown in mouse peritoneal macrophages, siRNA targeting mouse *Hif-1α*, *Hif-2α*, and control siRNA (Zorinbio, China) were prepared using Lipofectamine RNAiMAX Reagent (Thermo, USA) according to the manufacturer’s protocol. A final concentration of 10 μM siRNA was used.

### Quantitative reverse transcription PCR (qPCR)

qPCR was performed as previously described [25]. In brief, cells were lysed by RNAiso Plus (Takara, Japan) to collect total RNA, and cDNA was synthesized with RT reagent Kit with gDNA Eraser (Takara, Japan). A total of 1 μg cDNA was used for each qPCR reaction using TB Green (Takara, Japan) on an ABI Prism 7500 Sequence Detection system (Applied Biosystems). Fold change in mRNA expression was calculated with the ΔΔCT method using *Actb* as an endogenous control. All the expression data were finally calculated and shown as 2^−ΔΔCt^. Results are expressed as fold change normalized to the controls. The primers used were listed in Additional file 4: Table S1.

### Immunoblotting

Immunoblotting was performed as previously reported [26]. Briefly, the whole-cell lysates were prepared with RIPA containing 1% PMSF, protease, and phosphatase inhibitor cocktail (Thermo, USA) on ice. The protein concentration was measured with the BCA method (Beyotime, China). After being mixed with 5× loading buffer, the protein extracts were heated at 95 °C for 10 min. Immunoblotting was performed using antibodies against Transferrin receptor (Abcam, USA), HIF-1α (Cell Signaling, USA), and β-actin (Sigma, USA), serving as the internal reference. The membranes were exposed to the Tanon-5200 Chemiluminescent Image System (Tanon, China) for imaging.

### Immunofluorescent

For immunofluorescent staining of the tumor samples from the Hepa1-6 cell-implanted HCC model, tumors were dissected, embedded, and frozen for cryostat sectioning. The sections were fixed with 4% paraformaldehyde, permeabilized in 0.2% Triton X-100 and blocked with 5% goat serum, followed by staining with first antibodies against transferrin (Proteintech Group, China) and F4/80 (Thermo, USA) overnight. The next day, the samples were washed with TBST three times and stained with secondary antibodies conjugated with Alexa Fluor 488 and Alexa Fluor 594 at room temperature for 1 h. The cell nucleus was stained with DAPI. The samples were imaged by a fluorescence microscope. The co-localization of transferrin and F4/80 was determined using ImageJ software.

### T cell proliferation assay

The CD4^+^ and CD8^+^ T cells were magnetically enriched (Biolegend, USA) from the spleen of 6-week-old C57BL/6 mice. Separated T cells were seeded into 96-well plate pre-coated with 2 μg/mL anti-mouse CD3 (BioXcell, USA) in IMDM (Hyclone, USA) containing 2 μg/mL anti-mouse CD28 (BioXcell, USA), 10% fetal bovine serum, 100 U/mL interleukin-2 (R&D system, USA) and 2 mM l-glutamine (Gibco, USA). After being labeled with 1 μM CFSE (Thermo, USA), 3 × 10^6^ T cells were co-cultured with peritoneal macrophages with indicated treatment at a ratio of 2:1. After incubation for 3 days, cells were collected, and FACS analyzed the CFSE signal.

### Transferrin uptake assay

Mouse peritoneal macrophages were co-cultured with Hepa1-6/shNT or Hepa1-6/shTfrc cells in a 0.4 μm Transwell system. Cells were starved in serum-free DMEM for 24 h. After being washed with PBS three times, cells were incubated in serum-free DMEM containing 5 μM FITC-labeled holo-transferrin for 2 h. Next, the medium was removed, followed by imaging with a fluorescence microscope (Olympus, Japan) or FACS.

### Statistics

Statistical analysis was performed using the Prism software program (GraphPad 7 Software). Quantitative variables were analyzed by paired *t-*test. Data are presented as mean ± standard deviation (SD), repeated from three independent experiments. Statistical significance was set at *P* < 0.05 (two-sided).

## Results

### Ferrous iron is reduced in M2-like TAM from HCC patient samples and murine model

**Fig. 1:**
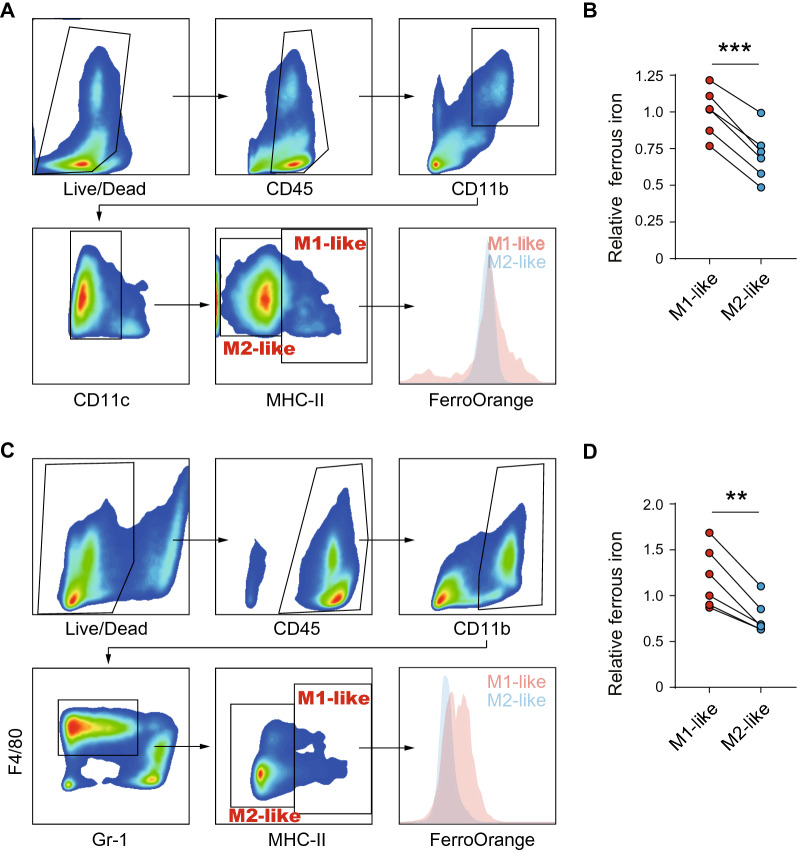
Ferrous iron is reduced in M2-like tumor-associated macrophages. **A** The sorting strategy of human M1- and M2-like tumor-associated macrophages from HCC tissues. **B** Ferrous iron was measured in human M1- and M2-like tumor-associated macrophages. **C** The sorting strategy of murine M1 and M2-like tumor-associated macrophages from HCC tissues. **D** Ferrous iron was measured in murine M1- and M2-like tumor-associated macrophages. Data from **B** and **D** were analyzed using paired *t-*test, **p < 0.01, ***p < 0.001

Ferrous iron is the primary source of intracellular iron that mediates the biological process. To investigate the role of iron in the functional polarization of TAM, we first compared the level of ferrous iron in M1-like (CD11b^+^CD11C^−^MHC-II^+^) and M2-like (CD11b^+^CD11C^−^MHC-II^−^) TAM in 6 fresh HCC tissue samples. FACS showed that ferrous iron in M2-like TAM was considerably decreased than in M1-like TAM (*p* < 0.001, Fig. 1A, B). We next established a murine HCC model to substantiate our findings in human samples. Similarly, M2-like TAM (CD11b^+^GR-1^−^F4/80^+^MHC-II^−^) exhibited decreased ferrous iron than M1-like TAM (CD11b^+^GR-1^−^F4/80^+^MHC-II^+^; Fig. 1C, D). These data indicate that ferrous iron is reduced in M2-like TAM from both human and murine HCC tissues, suggesting that iron may be involved in the functional polarization of TAM.

### Loss of iron in macrophages leads to immunosuppressive M2 polarization

**Fig. 2:**
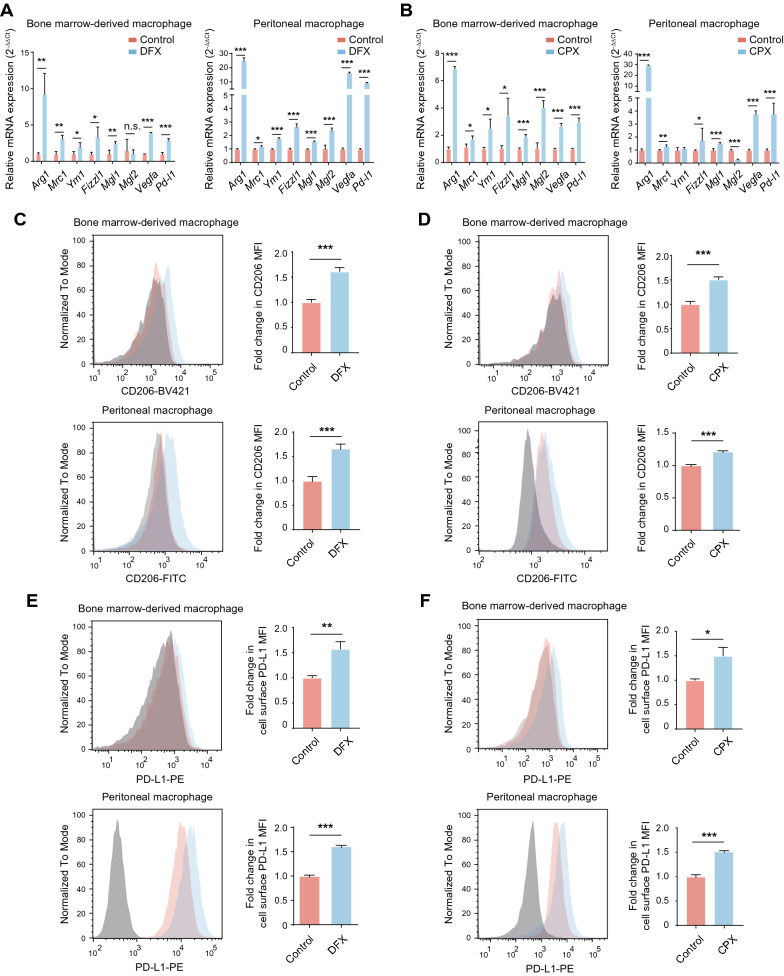
Iron deprivation drives the immunosuppressive polarization of macrophages. **A**, **B** The mRNA expression of M2 signature genes was detected in BMDMs and PMs with indicated treatment using qPCR. The results were shown as 2^−ΔΔCt^ and were expressed as relative fold change normalized to the controls. **C**–**F** The surface expression of CD206 and PD-L1 with indicated treatment in BMDMs and PMs using FACS. The results were shown as relative fold change in MFI of CD206 and PD-L1 normalized to the controls. All data are representative of three independent experiments and presented as mean ± SD. *p < 0.05, **p < 0.01, ***p < 0.001

Given that most TAM acquire an immunosuppressive M2-like phenotype and ferrous iron is reduced in M2-like TAM, we wondered whether iron loss contributes to M2-like TAM polarization. To this end, two iron chelators, deferasirox (DFX) and ciclopirox (CPX), were utilized to deprive iron from mouse bone marrow-derived macrophages (BMDMs) and peritoneal macrophages (PMs). Neither of the chelators showed cytotoxicity on both BMDMs and PMs (Additional file 1: Figure S1A). Both DFX and CPX upregulated the expression of M2 signature genes, including *Arg1*, *Vegfa*, and *Pd-l1* (Fig. 2A, B). However, M1 signature genes, including *iNOS*, *Cxcl9*, and *Cxcl10*, were unchanged (Additional file 1: Figure S1B). Likewise, FACS analysis showed that cell surface expression of inhibitory protein CD206 (also known as MRC1) and PD-L1 were elevated after DFX and CPX treatment (Fig. 2C–F). Similar findings were observed in murine RAW264.7 cells (Additional file 1: Figure S1C–G).

**Fig. 3:**
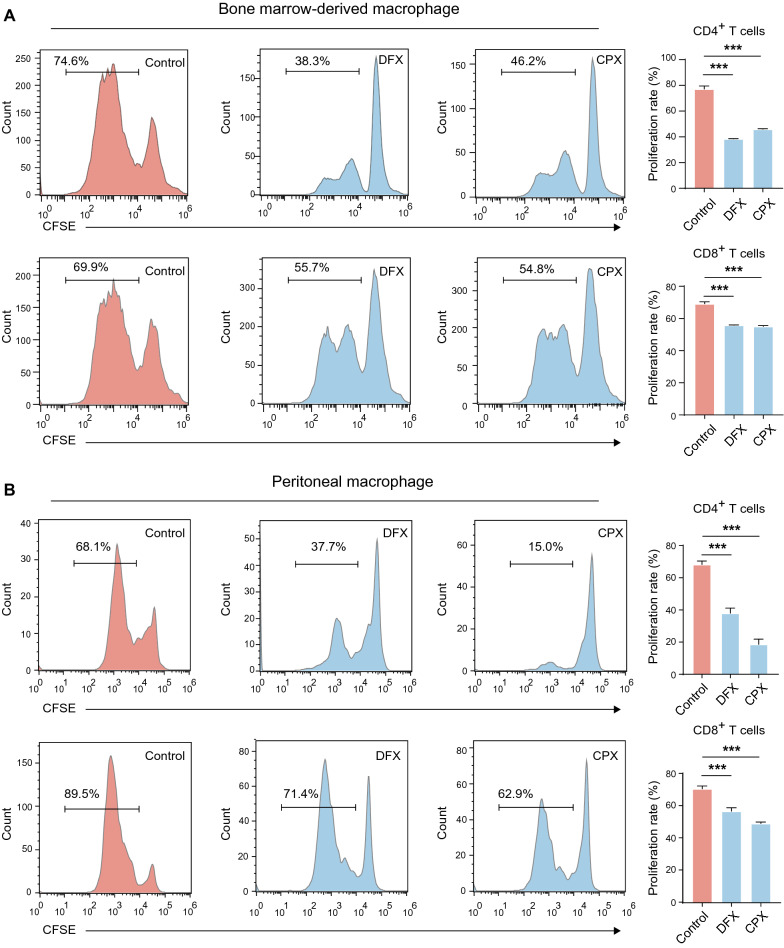
Iron deprivation drives the immunosuppressive function of macrophages. **A**, **B** CFSE-labeled murine spleen CD4^+^ and CD8^+^ T cells were co-cultured with BMDMs and PMs treated as described at a ratio of 1:2 for 3 days, and the fluorescence of CSFE was measured by FACS. The proliferation rate of T cells was determined as the proportion of cells with reduced CFSE intensity due to cell division. All data are representative of three independent experiments and presented as mean ± SD. *p < 0.05, **p < 0.01, ***p < 0.001

To further determine whether DFX- or CPX-treated macrophages were immunosuppressive, we performed a T cell proliferation assay by co-culturing CFSE-labeled CD4^+^ or CD8^+^ T cells with macrophages either treated with an iron chelator or left untreated. In this co-culture system, we found that the proliferation rates of CD4^+^ and CD8^+^ cells are significantly reduced when both BMDMs and PMs co-cultured with either DFX or CPX (Fig. 3A, B). Collectively, our data demonstrate that loss of iron increases M2 signature gene expression and leads to immunosuppressive polarization of macrophages.

### HCC cell-imposed iron restriction results in reduced ferrous iron in TAM and therefore drives their M2 polarization

**Fig. 4:**
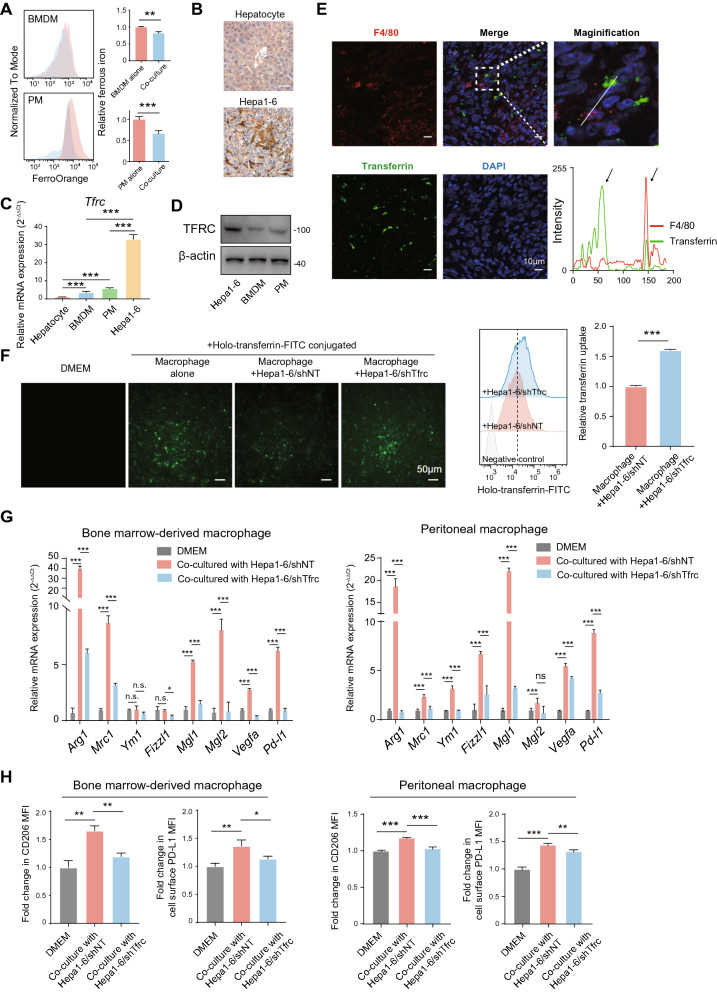
The TF/TFRC axis-mediated iron uptake by HCC cells competes for iron with macrophages and promotes M2 polarization. **A** BMDMs and PMs were co-cultured with indicated cells or DMEM alone, and the MFI of intracellular ferrous iron was determined by FACS. **B** Representative Immunohistochemistry staining of TFRC in tumor and non-tumor tissues from Hepa1-6-bearing mice. **C** *Tfrc* mRNA expression was detected in BMDMs, PMs, mouse hepatocytes, and Hepa1-6 cells by qPCR. The results were shown as 2^−ΔΔCt^ and were expressed as relative fold changes normalized to the controls. **D** The protein expression of TFRC was detected in Hepa1-6 cells, BMDMs, and PMs by Western blot. **E** Co-localization of TF and macrophage marker F4/80 was shown. The intensity profiles of TF and F4/80 along the white line were plotted. Arrows marked the positive signals. Green: TF, red: F4/80, and blue: DAPI. Scale bar, 10 μm. **F** PMs and Hepa1-6 cells in the co-culture system were allowed to grow in DMEM media containing 50 ng/ml FITC-labeled holo-transferrin, and then in vitro holo-transferrin uptake assay was performed. PMs were collected 2 h later, and the MFI of FITC-transferrin was measured by FACS. **G** The mRNA expression of M2 signature genes in BMDMs and PMs co-cultured with indicated cells or DMEM alone was detected using qPCR. The results were shown as 2^−ΔΔCt^ and were expressed as relative fold changes normalized to the controls. **H** Cell surface expression of CD206 and PD-L1 in BMDMs and PMs co-cultured with indicated cells or DMEM alone was determined by FACS. The results were shown as relative fold changes in MFI of CD206 and PD-L1 normalized to the controls. All data are representative of three independent experiments and presented as mean ± SD. **p < 0.01, ***p < 0.001

We next assumed that tumor cells are responsible for reduced ferrous iron in TAM. To this end, we first co-cultured macrophages with Hepa1-6 cells. The level of ferrous iron was significantly decreased in co-culturing BMDMs or PMs and Hepa1-6 cells, as indicated by FACS (Fig. 4A). A previous study demonstrated that breast tumor cells induce TAM into an iron-release phenotype [27]. We next examined the expression of genes that involves iron uptake (*Tfrc*), iron storage (*Fth1*), and iron export (*Slc40a1*) in macrophages. Surprisingly, macrophages co-cultured with Hepa1-6 cells but not AML-12 cells (a normal mouse liver cell line) significantly upregulated *Tfrc* while decreased *Fth1* and *Slc40a1*, suggesting a compensatory iron starvation response (Additional file 2: Figure S2A).

Next, we validated our findings in the GSE159254 dataset. When co-cultured with human HCC cell lines HepG2 or MHCC97H cells, THP-1 monocyte-derived macrophages upregulated genes involved in iron uptake (*ACO1*, *IREB2*, *TFRC*, and *SLC11A2*) and downregulated genes involved in iron storage and export (*TF*, and *SLC40A1*; Additional file 1: Figure S2B). These results ruled out the possibility that tumor cells educate PMs toward an iron-releasing phenotype.

Tumor cells and tumor-infiltrating lymphocytes compete for metabolic nutrients within the tumor niche [28]. We, therefore, asked whether iron competition between tumor cells and TAM leads to reduced ferrous iron in TAM. As the TF-TFRC axis is the central system that mediates cellular iron uptake [29], we assumed that HCC cells use the system for iron competition. To this end, we first examined TFRC levels in tumor tissues from Hepa1-6 tumor-bearing mice. We found that TFRC is mainly on tumor cells and normal hepatocytes, and Hepa1-6 cells had elevated TFRC levels compared to normal hepatocytes in our mouse HCC model (Fig. 4B).

Next, we estimated *Tfrc* expression in BMDMs, PMs, hepatocytes, and HCC cells. Although *Tfrc* expression in BMDMs and PMs was higher than that in hepatocytes, both mRNA and protein levels of TFRC in Hepa1-6 cells were far higher than that in BMDMs and PMs, suggesting that TF might be more likely to be absorbed by Hepa1-6 cells (Fig. 4C, D). These data indicate that Hepa1-6 cells compete with macrophages for iron by enforced TF/TFRC-mediated iron uptake. In support of this idea, we found that TF is not co-localized with TAMs in tumor tissue from Hepa1-6-bearing mice (Fig. 4E). In vitro holo-TF uptake assay further revealed that TF uptake by PMs is significantly reduced when co-culturing with Hepa1-6/shNT cells. In contrast, *Tfrc* knockdown in Hepa1-6 cells ameliorated this effect (Fig. 4F and Additional file 2: Figure S2C). These data support the idea that elevated TFRC in HCC cells might contribute to M2-like TAM polarization.

To further determine whether TFRC-mediated tumor cell-imposed iron restriction promotes M2 polarization of macrophages, we examined the expression of M2 signature genes in BMDMs and PMs co-cultured with either Hepa1-6/shTfrc cells or their controls. Both BMDMs and PMs co-cultured with Hepa1-6/shNT cells markedly increased the expression of M2 signature genes (Fig. 4G). In contrast, the increased expression of these genes was entirely or partially suppressed in BMDMs and PMs co-cultured with Hepa1-6/shTfrc cells (Fig. 4G). Similarly, cell surface CD206 and PD-L1 on BMDMs and PMs were elevated when co-culturing with Hepa1-6/shNT cells but reduced when co-culturing with Hepa1-6/shTfrc cells (Fig. 4H). To sum up, these results demonstrate that tumor cells make use of the TF-TFRC axis to restrict iron uptake by macrophages, leading to reduced ferrous iron levels and M2 polarization in macrophages.

### Iron deprivation drives M2 polarization of macrophage through HIF-1α-dependent manner

**Fig. 5:**
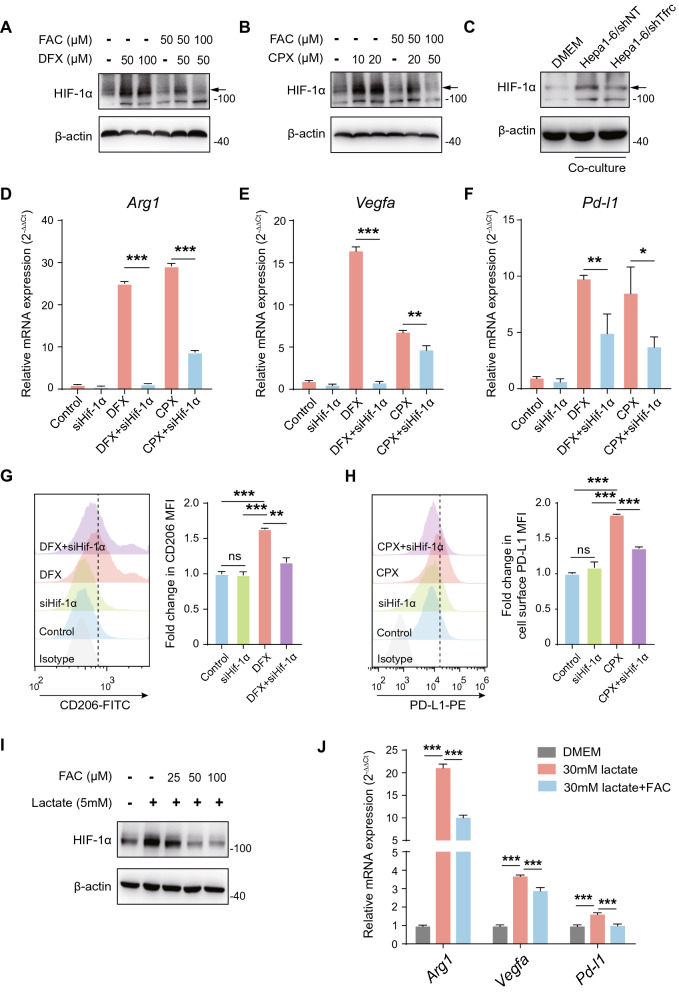
Iron deprivation drives M2 polarization of macrophage through induction of HIF-1α. **A**, **B** The protein expression of HIF-1α in PMs with indicated treatment by Western blot. **C** The protein expression of HIF-1α in PMs co-cultured with indicated cells or DMEM alone by Western blot. **D**–**F** The mRNA expression of *Arg1*, *Vegfa*, and *Pd-l1* by qPCR in PMs with either *Hif-1α* knockdown or its control counterpart upon indicated treatment. The results were shown as 2^−ΔΔCt^ and were expressed as relative fold changes normalized to the controls. **G**, **H** Cell surface expression of CD206 and PD-L1 by FACS in PMs with either *Hif-1α* knockdown or its control counterpart upon indicated treatment. The results were shown as relative fold changes in MFI of CD206 and PD-L1 normalized to the controls. **I** The protein expression of HIF-1α in PMs treated as described. **J** The mRNA expression of *Arg1*, *Vegfa*, and *Pd-l1* was determined by qPCR in PMs treated as described. The fold changes in expression level relative to control were expressed as 2^−ΔΔCt^. All data are representative of three independent experiments and presented as mean ± SD. *p < 0.05, **p < 0.01, ***p < 0.001

HIF-1α and HIF-2α are critical nuclear transcriptional factors that regulate the cellular response to hypoxia and contribute to the functional polarization of macrophages. We found that the mRNA expression of *Hif-1α* and *Hif-2α* remains unaltered (Additional file 3: Figure S3A, B), while the protein expression of HIF-1α, but not HIF-2α, was increased under DFX treatment in PMs (Fig. 5A). In contrast, treatment with ferric citrate (FAC) reduced HIF-1α protein expression and inhibited increased HIF-1Α resulting from DFX treatment in a dose-dependent manner in PMs (Fig. 5A). A similar effect was also found in CPX-treated PMs (Fig. 5B). Nevertheless, we did not detect altered HIF-2α upon either DFX or CPX treatment (Additional file 2: Figure S2C). Additionally, although co-culturing with Hepa1-6 cells significantly induced HIF-1α expression in PMs, co-culturing with Hepa1-6/shTfrc cells could partially reverse this process (Fig. 5C). These data suggest that loss of iron can induce HIF-1α protein expression in macrophages.

To further investigate whether increased HIF-1α-mediated M2 polarization of PMs in the context of iron deprivation, we utilized specific *Hif-1α*-targeting siRNA to knockdown *Hif-1α* in PMs under either DFX or CPX treatment. DFX or CPX significantly upregulated *Arg1*, *Vegfa*, and *Pd-l1* expression, while knockdown of *Hif-1α* entirely or partially blocked the up-regulation (Fig. 5D–F). Consistently, *Hif-1α* knockdown could rescue the induction of cell surface CD206 and PD-L1 under DFX or CPX treatment (Fig. 5G, H). However, knockdown of *Hif-2α* had no effect, suggesting that Hif-2α might not involve in this process (Additional file 2: Figure S2D). It has been demonstrated that tumor-derived lactate is a potent inducer of HIF-1α in TAM (10). To further support our findings, we tested whether iron could affect HIF-1α induction in PMs upon lactate treatment. As expected, lactate was sufficient to induce HIF-1α expression in PMs. However, FAC treatment could inhibit lactate-induced HIF-1α expression in a dose-dependent manner (Fig. 5I). Moreover, FAC treatment could also partially block lactate-induced mRNA expression of *Arg1*, *Vegfa*, and *Pd-l1* (Fig. 5J). Notably, the concentration of lactate was equivalent in the supernatant of Hepa1-6/shTfrc cells and its control counterpart (Additional file 3: Figure S3E). Together, these results demonstrate that iron deprivation drives macrophages towards M2 polarization by increased HIF-1α expression.

### TFRC expression is elevated and associated with M2 macrophage infiltration in HCC patients

**Fig. 6:**
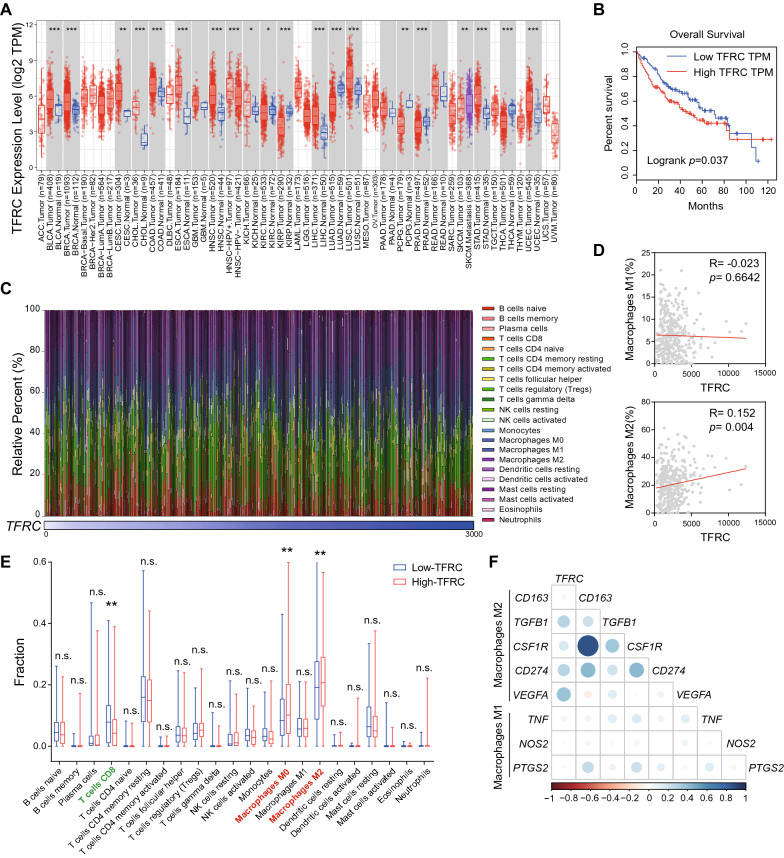
*TFRC* is associated with suppressive tumor immune microenvironment and poor prognosis for HCC. **A** *TFRC* mRNA expression in 33 types of TCGA tumors. **B** Kaplan–Meier curves for overall survival stratified according to high and low TFRC expression in TCGA LIHC database. **C** The relative percent of 22 types of immune cells in each LIHC sample (n = 366). **D** Scatter plot showing positive correlation of TFRC expression and infiltration level of M2-macrophages but not M1-macrophages in TCGA LIHC database. **E** The fraction of 22 types of immune cells in the Low- and High-TFRC group, respectively. Median TFRC expression serves as the cut-off to divide HCC patients into Low- and High-TFRC groups. **F** Correlation between TFRC and M1/M2 signature genes. Color depth and circle square represent the degrees of correlation. **p < 0.01, ***p < 0.001

Finally, we validated our findings in TCGA database. To this end, we analyzed *TFRC* mRNA expression based on the multi-cancer cohort data obtained from TCGA using the TIMER. We found that *TFRC* mRNA expression was elevated in 20 types of tumor, including breast invasive carcinoma (BRCA), colon adenocarcinoma (COAD), LIHC, and lung adenocarcinoma (LUAD) as compared to normal tissues, providing that upregulated *TFRC* is a common feature in cancers (Fig. 6A). It ought to be noted that HCC patients with high TFRC levels have significantly shortened overall survival (Fig. 6B).

To determine whether TFRC expression correlated with HCC immune microenvironment, we algorithmically analyzed the diversity and landscape of 22 tumor-infiltrating immune cells using the CIBERSORT database (Fig. 6C). The results showed that *TFRC* mRNA expression in HCC patients was positively related to the relative percent of tumor-infiltrating M2 macrophages but not M1 macrophages (Fig. 6D). We further divided LIHC patients into Low-TFRC and High-TFRC subgroups using median *TFRC* expression as the cut-off. We found patients with high *TFRC* expression had a higher fraction of infiltrating M2 macrophages than those with low *TFRC* expression. In contrast, patients with high *TFRC* expression had lower infiltrating CD8^+^ T cells than those with low *TFRC* expression (Fig. 6E). Moreover, *TFRC* had a significant positive correlation with the expression of M2 signature genes (*CD163*, *TGFB1*, *CSF1R*, *CD274*, and* VEGFA*), but not the expression of M1 marker genes (*TNF*, *NOS2*, and* PTGS2*; Fig. 6F). Collectively, these data support our in vitro results and provide evidence that TFRC is associated with M2 macrophage infiltration and poor prognosis in HCC.

## Discussion

The polarization of macrophages is regulated by a complex biologic network. Previous studies have revealed the regulation role of iron in macrophage polarization. Iron supplementation has been shown to confer pro-inflammatory M1 polarization of macrophages [30]. Dietary iron overload of mice can lead to hepatic M1 macrophage polarization [24]. In the context of wound healing, however, iron from tissue promotes M2 macrophage polarization [31]. Another study demonstrates that chronic iron overload promotes M2-like polarization of THP-1 monocyte-derived macrophages [32]. These seemingly contradictory results highlight a context- and tissue-dependent macrophage polarization. M2 macrophages, which resemble tumor-associated macrophages, express iron-efflux genes with increased *SLC40A1* and decreased *ferritin* [33]. When cultured with breast cancer cell lines, macrophages upregulate *SLC40A1* and *LCN2* to acquire an iron-releasing phenotype to support cancer proliferation [27]. These results suggest that macrophages tend to reduce intracellular iron levels in the context of cancer, which contributes to the M2-like polarization of TAM. In this study, we revealed that ferrous iron, an active form of iron, was significantly reduced in M2-like TAM when compared to M1-like TAM. Interestingly, we also found *Slc40a1* and *Fth1* were decreased while *Tfrc* was increased when TAM was co-cultured with HCC cells. These findings suggested that HCC cells do not directly educate macrophages toward an iron-releasing phenotype but reduce the iron level in macrophages through other mechanisms.

It is well established that iron metabolism is frequently dysregulated across varieties of solid human malignancies [34]. Tumor cells have a high demand for iron during their uncontrolled proliferation, and thus they acquire elevated iron uptake profile or lowered iron efflux profile. Both a low iron uptake (TFRC^low^HFE^high^) and a high iron efflux (SLC40A1^high^hepdicin^low^) profile confer a favorable prognosis of patients with breast cancer [35]. However, the potential relationship between tumor iron metabolism and non-tumoural component within TME is largely unknown. Indeed, metabolic crosstalk between cancer cells and tumor-infiltrating lymphocytes (TIL) plays an essential role in forming an immunosuppressive tumor niche. Chang et al*.* reported that tumor cells and TIL compete for glucose within TME [28]. Glucose consumption by tumor cells can metabolically restrict T cells, impairing their effector function, and accelerating tumor progression. Although previous studies focus on the effects of iron metabolism on the tumor itself, we identified, in the current study, that tumor cells overexpressed TFRC and could directly compete for iron with tumor-associated macrophages in the context of HCC, leading to immunosuppressive M2 polarization of macrophages. Our findings revealed how tumor iron metabolism affected iron-handling property and functional polarization of TAM, providing new interconnections between tumor-intrinsic iron metabolism and tumor immunity.

Hypoxia, identified as a negative factor related to poor prognosis and resistance to anti-cancer therapy, is a hallmark of the tumor niche owing to rapid cell division and increased tumor angiogenesis [36]. HIF-1α and HIF-2α, as most studied members of HIFs, are broadly expressed in varieties of tumor types. Given the critical role of HIFs in the functional polarization of TAM, we herein revealed that iron deprivation mainly drives M2 polarization of macrophages through induction of HIF-1α but not HIF-2α. Although HIF-1α and HIF-2α have overlapping functions, we found that HIF-1α acts as a significant mediator in iron-induced macrophage polarization, suggesting that some unique roles performed by these two transcriptional factors still need to be further explored. Notably, the hypoxic activity of HIFs is mainly dependent on protein stabilization of HIF-1α and HIF-2α [37]. It has been concluded that HIFs are post-transcriptionally controlled by prolyl hydroxylase domain (PHD) family-mediated proteasomal degradation. Importantly, ferrous iron is essential for PHD hydroxylase activity [37]. We observed that iron deprivation induced HIF-1α protein expression while its mRNA expression remained unchanged, which indicated that loss of iron might stabilize HIF-1α by reducing PHD hydroxylase activity. Tumor-derived lactate is a potent inducer of HIF-1α, and lactate promotes immunosuppressive polarization of TAM in a HIF-1α-dependent manner [10]. Our data revealed that iron antagonizes M2 macrophage polarization induced by lactate, further supporting the conclusion that the regulation effect of iron on macrophage polarization is mediated by HIF-1α.

In conclusion, our study demonstrated that cancer cell-imposed iron restriction drives M2 immunosuppressive polarization of macrophages contributing to inhibition of anti-tumor immunity. Our data also provide new interconnections between tumor-intrinsic iron metabolism and suppressive tumor immune microenvironment through the TF/TFRC axis. These novel findings identify tumor TFRC as a valuable target for cancer immunoprevention and immunotherapy.

## Supplementary Information

**Additional file 1: Figure S1.** Iron deprivation drives the immunosuppressive polarization of macrophages. (A) Cell viability was measured in BMDMs and PMs with indicated treatment by CCK-8 assay. (B) The mRNA expression of M1 signature genes in PMs with indicated treatment using qPCR. The fold changes in expression level relative to control were expressed as 2^−ΔΔCt^. (C) The mRNA expression of M2 signature genes in RAW264.7 cells with indicated treatment using qPCR. The fold changes in expression level relative to control were expressed as 2^−ΔΔCt^. (D–G) The surface expression of CD206 and PD-L1 with indicated treatment in RAW264.7 cells using FACS. The results were shown as relative fold changes in MFI of CD206 and PD-L1 normalized to their corresponding controls. All data are representative of three independent experiments and presented as mean ± SD. *p < 0.05, **p < 0.01, ***p < 0.001.

**Additional file 2: Figure S2.** Tumor cells fail to educate macrophages toward an iron-releasing phenotype but instead an iron starvation response. (A) The mRNA expression of *Tfrc*, *Fth1*, and *SLC40A1* in PMs co-cultured with indicated cells or DMEM alone using qPCR. The fold changes in expression level relative to control were expressed as 2^−ΔΔCt^. (B) Heat map of iron metabolism-related genes in THP-1 monocyte-derived macrophages based on RNA-seq data from GSE159254. (C) BMDMs and Hepa1-6 cells in the co-culture system were allowed to grow in DMEM media containing 50 ng/mL FITC-labeled holo-transferrin, and then in vitro holo-transferrin uptake assay was performed. BMDMs were collected 2 h later, and the MFI of FITC-transferrin was measured by FACS. All data are representative of three independent experiments and presented as mean ± SD. *p < 0.05, ***p < 0.001.

**Additional file 3: Figure S3.** Iron deprivation drives macrophage M2 polarization through induction of Hif-1α. (A, B) The mRNA expression of *Hif-1α* and *Hif-2α* in PMs with indicated treatment using qPCR. The fold changes in expression level relative to control were expressed as 2^−ΔΔCt^. (C) The protein expression of HIF-2α in PMs with indicated treatment was detected by Western blot. (D) The mRNA expression of *Arg1*, *Vegfa*, and *Pd-l1* by qPCR in BMDMs with either *Hif-2α* knockdown or its control counterpart upon indicated treatment. The fold changes in expression level relative to control were expressed as 2^−ΔΔCt^. (E) The concentration of lactate in the supernatants of Hepa1-6/shNT and Hepa1-6/shTfrc cells was measured by lactate assay kit.

**Additional file 4: Table S1.** List of primers used for qPCR analysis in this study.

## Data Availability

The datasets used and analyzed during the current study are available from the corresponding author on reasonable request.

## Abbreviations

ARG1: Arginase-1
BRCA: Breast invasive carcinoma
CPX: Ciclopirox
COAD: Colon adenocarcinoma
DFX: Deferasirox
FACS: Flow cytometry
GEPIA: The Gene Expression Profiling Interactive Analysis
HCC: Hepatocellular carcinoma
LIP: Labile iron pool
LCN2: Lipocalin-2
LUAD: Lung adenocarcinoma
OS: Overall survival
PD-L1: Programmed cell death-ligand
qPCR: Quantitative reverse transcription PCR
SLC40A1: Solute carrier family 40 member 1
TAM: Tumor-associated macrophages
TCGA: The Cancer Genome Atlas
TFRC: Transferrin receptor
TME: Tumor microenvironment
TIMER: Tumor Immune Estimation Resource
VEGFA: Vascular endothelial growth factor type A
