# Supplementary figures and images for: Tumor cell-imposed iron restriction drives immunosuppressive polarization of tumor-associated macrophages
Source: J Transl Med. 2021 Aug 13;19:347. doi: 10.1186/s12967-021-03034-7 (PMC8361643; doi:10.1186/s12967-021-03034-7)

**A**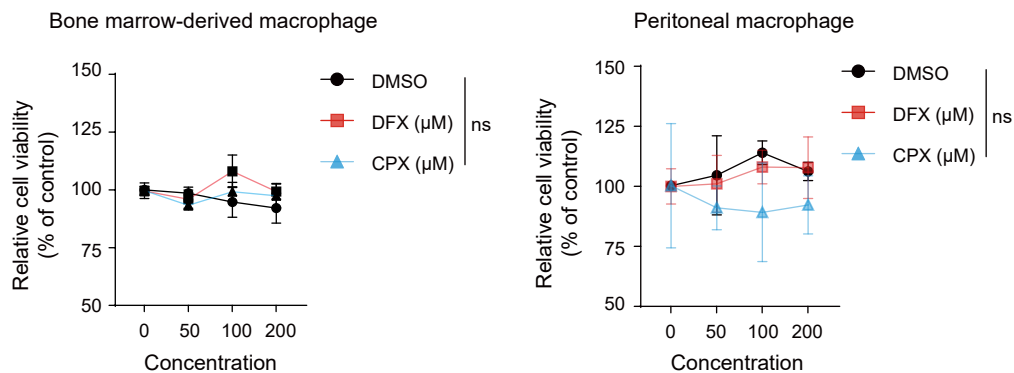**B**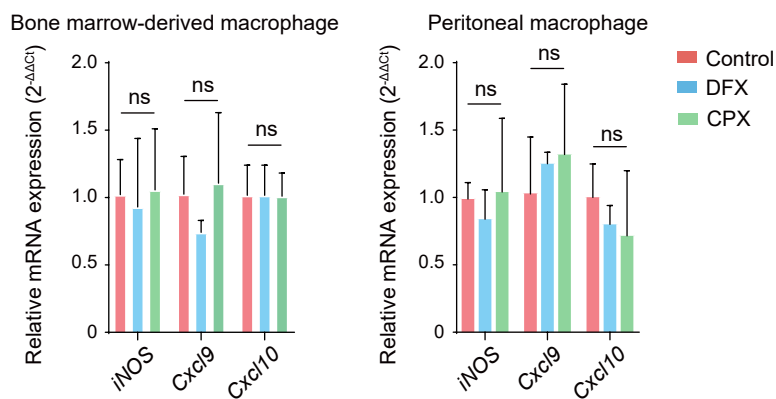**C**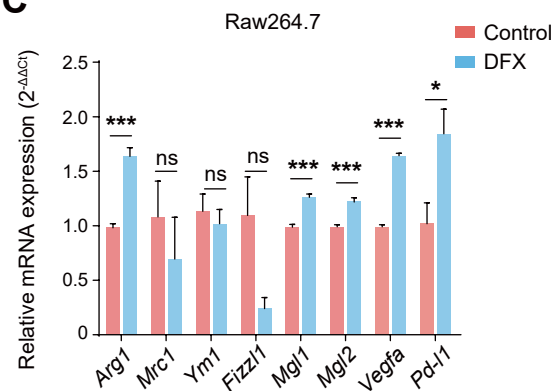**D**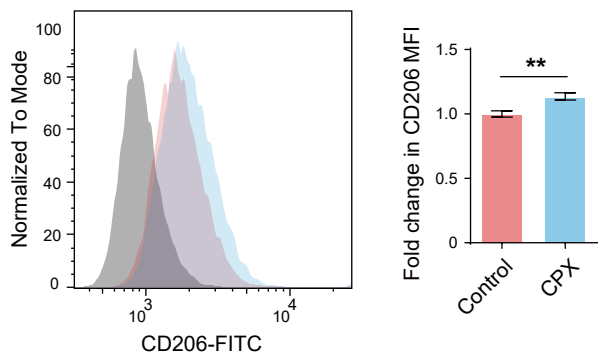**E**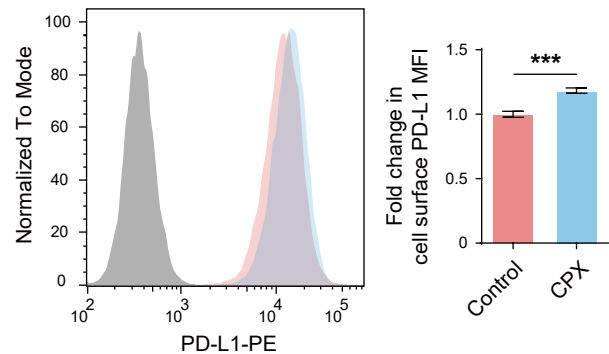**F**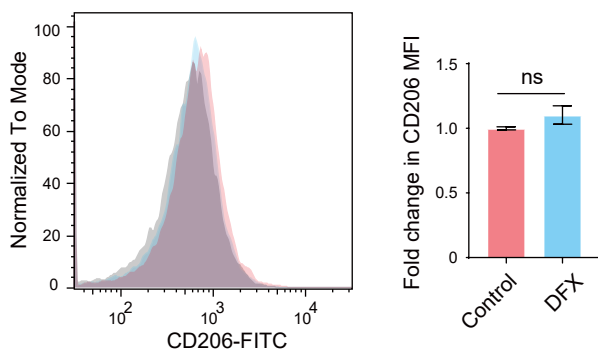**G**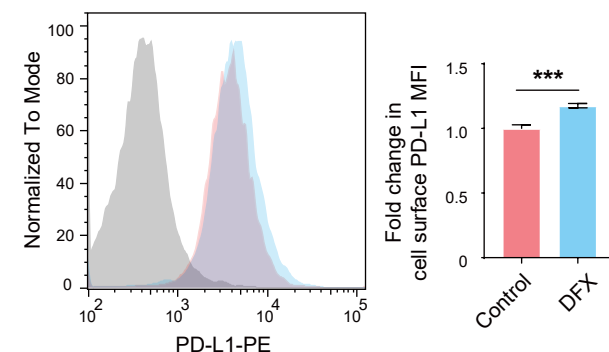

Supplement: Supplementary file 1 — Additional file 1: Figure S1. Iron deprivation drives the immunosuppressive polarization of macrophages. (A) Cell viability was measured in BMDMs and PMs with indicated treatment by CCK-8 assay. (B) The mRNA expression of M1 signature genes in PMs with indicated treatment using qPCR. The fold changes in expression level relative to control were expressed as 2−ΔΔCt. (C) The mRNA expression of M2 signature genes in RAW264.7 cells with indicated treatment using qPCR. The fold changes in expression level relative to control were expressed as 2−ΔΔCt. (D–G) The surface expression of CD206 and PD-L1 with indicated treatment in RAW264.7 cells using FACS. The results were shown as relative fold changes in MFI of CD206 and PD-L1 normalized to their corresponding controls. All data are representative of three independent experiments and presented as mean ± SD. *p < 0.05, **p < 0.01, ***p < 0.001. [file 12967_2021_3034_MOESM1_ESM.pdf]

**A**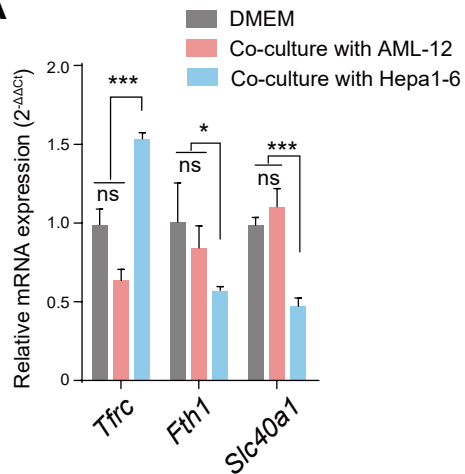**B**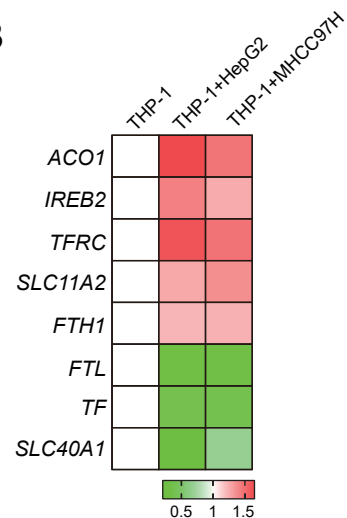**C**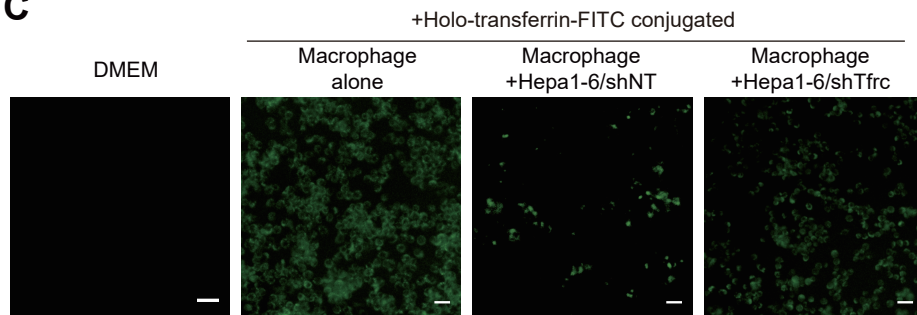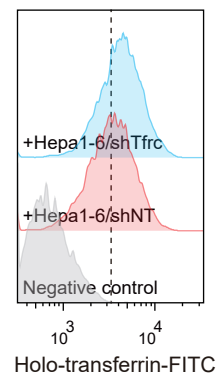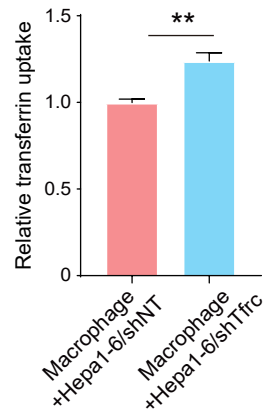

Supplement: Supplementary file 2 — Additional file 2: Figure S2. Tumor cells fail to educate macrophages toward an iron-releasing phenotype but instead an iron starvation response. (A) The mRNA expression of Tfrc, Fth1, and SLC40A1 in PMs co-cultured with indicated cells or DMEM alone using qPCR. The fold changes in expression level relative to control were expressed as 2−ΔΔCt. (B) Heat map of iron metabolism-related genes in THP-1 monocyte-derived macrophages based on RNA-seq data from GSE159254. (C) BMDMs and Hepa1-6 cells in the co-culture system were allowed to grow in DMEM media containing 50 ng/mL FITC-labeled holo-transferrin, and then in vitro holo-transferrin uptake assay was performed. BMDMs were collected 2 h later, and the MFI of FITC-transferrin was measured by FACS. All data are representative of three independent experiments and presented as mean ± SD. *p < 0.05, ***p < 0.001. [file 12967_2021_3034_MOESM2_ESM.pdf]

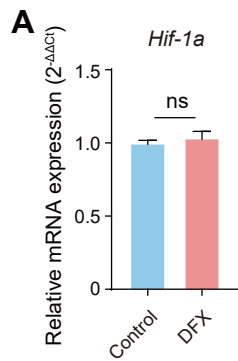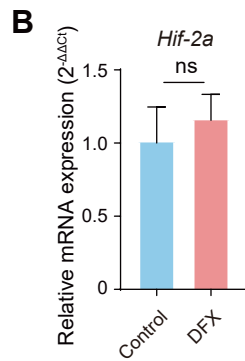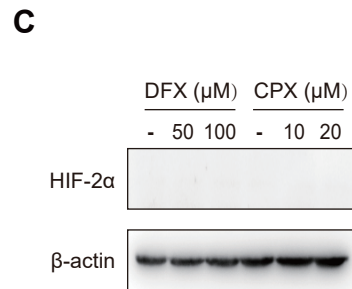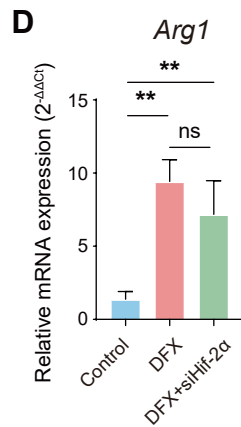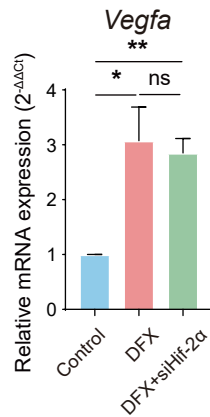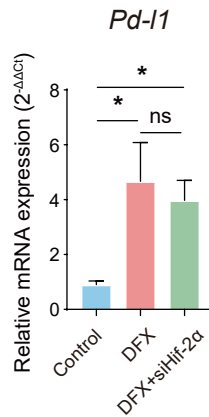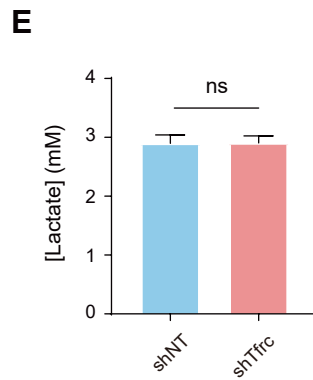

Supplement: Supplementary file 3 — Additional file 3: Figure S3. Iron deprivation drives macrophage M2 polarization through induction of Hif-1α. (A, B) The mRNA expression of Hif-1α and Hif-2α in PMs with indicated treatment using qPCR. The fold changes in expression level relative to control were expressed as 2−ΔΔCt. (C) The protein expression of HIF-2α in PMs with indicated treatment was detected by Western blot. (D) The mRNA expression of Arg1, Vegfa, and Pd-l1 by qPCR in BMDMs with either Hif-2α knockdown or its control counterpart upon indicated treatment. The fold changes in expression level relative to control were expressed as 2−ΔΔCt. (E) The concentration of lactate in the supernatants of Hepa1-6/shNT and Hepa1-6/shTfrc cells was measured by lactate assay kit. [file 12967_2021_3034_MOESM3_ESM.pdf]
