# Supplementary material for: Tumor cell-imposed iron restriction drives immunosuppressive polarization of tumor-associated macrophages
Source: J Transl Med. 2021 Aug 13;19:347. doi: 10.1186/s12967-021-03034-7 (PMC8361643; doi:10.1186/s12967-021-03034-7)
Supplement: Supplementary file 4 — Additional file 4: Table S1. List of primers used for qPCR analysis in this study. [file 12967_2021_3034_MOESM4_ESM.docx]

**Table S1. PCR primers**

| Gene | Forward primer | Reverse primer |
| --- | --- | --- |
| *Pd-l1* | GCTCCAAAGGACTTGTACGTG | TGATCTGAAGGGCAGCATTTC |
| *Fizzl1* | CCTGCTGGGATGACTG CTA | TGGGTTCTCCACCTCTTCAT |
| *Mgl1* | CAGAATCGCTT AGCCAATGTGG | TCCCAGTCCGTGTCCGAAC |
| *Mgl2* | T TCAAGAATTGGAGGCCACT | CAGACATCGTCATTCCAACG |
| *Mrc1* | AAGGCTATCCTGGTGGAAGAA | AGG GAAGGGTCAGTCTGTGTT |
| *Vegfa* | CCACGACAGAAGGAGAGCAGAAGTCC | C GTTACAGCAGCCTGCACAGCG |
| *Ym1* | GCCACTGAGGTCTGGGA TGC | TCCTTGAGCCACTGAGCCTTC |
| *Tfr1* | AGTGTCAGAAAACCCAAGAGG | CGTTTCAGCCAGTTTCACAC |
| *Arg1* | CCACAGTCTGGCAGTTGGAAG | GGTTGTCAGGGGAGTGTTGATG |
| *Hif1a* | TCTCGGCGAAGCAAAGAGTC | AGCCATCTAGGGCTTTCAGATAA |
| *Hif2a* | CACTGGCCCATGTCTTCCAT | GTGTTGGATCTGCCTCCCAT |
| *Slc40a1* | CTCCAACCCGCTCCCATAAG | AGCCTTATGCCGAAAGACCC |
| *Fth1* | AACAGTGCTTGAACGGAACC | GTGGTAGTTCTGGCGCACTT |
